# Zuogui Jiangtang Jieyu Formulation Prevents Hyperglycaemia and Depressive-Like Behaviour in Rats by Reducing the Glucocorticoid Level in Plasma and Hippocampus

**DOI:** 10.1155/2015/158361

**Published:** 2015-07-27

**Authors:** YuHong Wang, Hui Yang, Wei Li, Pan Meng, YuanShan Han, Xiuli Zhang, DeLiang Cao, Yuansheng Tan

**Affiliations:** ^1^Hunan University of Chinese Medicine, No. 300, Bachelor Road, Changsha, Hunan 410208, China; ^2^First Hospital of Hunan University of Chinese Medicine, Hunan, China

## Abstract

*Aim*. To determine whether Zuogui Jiangtang Jieyu prescription (ZGJTJY) has hypoglycemic and antidepressant effects which are mediated by corticosterone through adjustment of 11*β*-hydroxysteroid dehydrogenase type 1 (11*β*-HSD1) and glucocorticoid (GR) levels.* Materials and Methods*. The diabetes-related depression rats were randomly divided into four groups: the model group, metformin (1.8 mg/kg) combined with fluoxetine (10.8 mg/kg) group, and ZGJTJY high and low dose groups. Four weeks after modeling, blood glucose, behavior, and cognitive function of depression were detected. The expressions of 11*β*-HSD1 and GR in hippocampus were measured by western blotting and immunohistochemical experiments.* Results*. We found that (1) the treatment with ZGJTJY (10.26 g/kg) increases the motor activities and improves cognition ability. (2) ZGJTJY (10.26 g/kg) significantly relieves the disorder in blood and the relative indexes. (3) ZGJTJY (10.26 g/kg) can reduce hippocampal corticosterone expression levels and further improve hippocampus pathological changes. (4) ZGJTJY increased the expression of GR accompanied with decreasing 11*β*-HSD1 in hippocampus.* Conclusions*. ZGJTJY inhibits the expression of 11*β*-HSD1 and increases GR in hippocampus and subsequently modulates blood glucose levels, and therefore it is potential property that ZGJTJY could be of benefit for the treatment of behavior and cognitive function of diabetes-related depression.

## 1. Introduction

Diabetes, a global chronic disease, affects almost 382 million people [1]. Although a stable blood glucose can reduce the risk of complications occurring and reduce the impact on patients' normal life [2], it is unfortunately that a well-controlled blood glucose is not the key point that helps to stop the central erosion which is caused by diabetes mellitus. This erosion not only worsens the physical condition and quality of life [3], but also is regarded as the major mortality predictor in patients [4, 5].

It is regretful to mention that the research of type 2 diabetes-related depression is usually assessed in terms of clinical aspect. In the past decade, the experimental research is barely related to the treatment of diabetes with depression, except a study presented in 2014 that evaluated the therapeutic effects of Zuogui Jiangtang Jieyu prescription on the aspects of glucose and behavioral activity [6]. Although this paper is important from an experimental standpoint, a further investigation seems to be necessary at present. Hippocampus is recognized as not only the first tissue that is affected by diabetes mellitus, but also the major part that related to depression [7, 8]. We hypothesized that the protective effect of Zuogui Jiangtang Jieyu prescription in hippocampus is an important contributor to its hypoglycemic and antidepression function.

It is common to improve the classical Chinese prescription through the combination with characters of modern disease to adapt the illness in China. Zuogui Jiangtang Jieyu Fang, a prescription based on the characteristics of diabetes-related depression, is a Chinese herbal prescription on the strength of Zuogui Wan which has a long history in reducing blood glucose and it was listed in the Chinese authority “Pharmacopoeia” and developed by Jingyue Zhang during Ming dynasty. Zuogui Jiangtang Jieyu prescription including* Astragalus*,* Hypericum perforatum*, cooked* Rehmannia*, cornel, medlar, dodder,* Eucommia ulmoides*,* Salvia miltiorrhiza*, cortex moutan, and radix achyranthis bidentatae and added to turmeric and hyperforin perforatum which have hypoglycemic and antidepressant effects significantly. Various researches indicated that some Chinese medicine herbs including* Astragalus* polysaccharide, corni fructus, and* Achyranthes aspera* are efficiently improving the glucose homeostasis through a variety of pathways [9–11]. Zhang et al. [12] demonstrated that curcumin, a natural polyphenolic compound of* Curcuma longa*, had an antidepression effect by increasing brain-derived neurotrophic factor (BDNF) which is one of the underlying mechanisms in treatment. Tian et al. [13] have studied adhyperforin, a novel constituent of* Hypericum perforatum* L. The effects of inhibited uptake of serotonin, norepinephrine, dopamine, and displayed robust binding affinities for the serotonin and norepinephrine transporters provide the first evidence for* Hypericum perforatum* L.'s antidepressant-like activity.

Recent researches suggest that the biological bases of diabetes and depression are related to HPA axis disorder and then the disorder causes an abnormal increase of cortisol (in human) and corticosterone (CORT in rodents) [14–17], which are easily penetrated into the blood-brain barrier and damage the nerves. Previous studies found that there are two essential proteins, 11*β*-HSD1 and GR, expressed in hippocampus and closely related to CORT. The functions of these two proteins include CORT activation and stimulation of HPA axis negative feedback activity, and they are activated by binding to CORT, respectively [14, 18, 19]. Thus, a partial increase of CORT and damaged hippocampal neuron usually attribute to the disordered expression of 11*β*-HSD1 and GR [19, 20]. In addition, hippocampus is a key target organ that regulates emotion and cognitive function, which is essential for occurrence of depression [8]. Therefore, we believe that hyperactivity of HPA axis in diabetes contributes to an abnormal high level of CORT and it has been overtransferred to hippocampus. Moreover, a deregulated 11*β*-HSD1 leads to the CORT hyperactivity. Meanwhile, decreased expression of GR leads to a situation that CORT fails to cause negative feedback and regulate the HPA axis disorders. And it causes CORT accumulation in hippocampus, which injures neurons and induces diabetes-related depression.

In order to test the above hypothesis, we intend to verify ZGJTJY on the treatment of diabetes-related depression from the aspects of ethology and hematology. And then the expression of 11*β*-HSD1 and GR in the hippocampus was detected through immunohistochemistry and western blotting, while determining the content of CORT in the hippocampus by ELISA. Finally, hippocampal morphology was observed through HE stain. In addition, the purpose of this study is to determine the reasons of the abnormal increase of CORT in hippocampus in rats with diabetes-related depression, as well as neuronal damage, and verify the therapeutic effect of ZGJTJY.

## 2. Materials and Methods

### 2.1. Drugs and Reagent

The raw material of ZGJTJY was purchased and concentrated to oral liquid (1.14 g/mL) in the First Hospital of Hunan University of Chinese Medicine. The prescription consists of 18 g* Astragalus*, 3 g* Hypericum perforatum*, 9 g turmeric, 15 g cooked* Rehmannia*, 12 g cornel, 12 g medlar, 9 g dodder, 9 g* Eucommia ulmoides*, 12 g* Salvia miltiorrhiza*, 6 g cortex moutan, and 9 g radix achyranthis bidentatae. Metformin hydrochloride tablets (0.25 g) and fluoxetine hydrochloride capsules (20 mg) were purchased from Hunan Xiangya Pharmaceutical and Patheon, France, respectively. High fat diet consists of 10% cholesterol, 0.2% propylthiouracil, 20% lard oil, 20% Tween 80, and 20% propylene glycol and then is added to still water to 100 mL.

### 2.2. Animal Materials

75 male SD rats weighing 180–200 g were obtained from Hunan Province Slack Scene of Laboratory Animal Company and kept in SPF Laboratory Animal Center in Hunan Chinese Medicine University. The experiment has been ethically acceptable and where relevant conforms to the national guidelines for animal usage in the research.

### 2.3. Rats Molding

10 mL/kg high fat diet was given by intragastric injection for 14 days except control group. After that, abrosia with water supply was performed for 24 h before 38 mg/kg streptozotocin (Sigma-Aldrich Co., USA) injection on the tail. Control group was infused with 2 mL/kg 0.1 mol/L citrate buffer instead. After 72 h, fast blood glucose was detected to screen the diabetes rats. And then, the screened rats were exposed to 28 days of unpredictable chronic mild stress (UCMS) including (I) 4°C ice water bath (5 min), (II) 45°C hot stimulus (5 min), (III) pour cage 45°C (24 h), (IV) noise (8 h), (V) day and night upside down (24 h), (VI) damp bedding (200 mL/cage, 24 h), and (VII) clip tail (1 min). The stress was performed once per day randomly.

After molding, diabetes-related depression rats were divided into five groups consisting of vehicle, DMGB/F (treated with 1.8 mg/kg DMGB and 10.8 mg/kg fluoxetine), ZGJTJY/H, and ZGJTJY/L (treated with gradient concentration of Zuogui Jiangtang Jieyu by 2.28 g/m and 0.57 g/mL, resp.). In addition, control group was given equal volume of normal saline instead.

### 2.4. Open Field Test

Open field test was carried out in an 80 cm × 80 cm × 40 cm open field chamber. The floor of the chamber was divided into 25 equilateral squares. The horizontal movement (four feet within a square counted as one score) and vertical movement (two front paws to vacate counted as one score) were counted within 3 min after 1 min adaptation. The test was taken once every week.

### 2.5. Morris Water Maze Test

Spatial learning and memory were tested by Morris water maze. The Morris water maze was filled with water and divided into four quadrants. There was an underwater platform placed in one of the quadrants. After that, in the place navigation test, the time for rats to locate the underwater platform was regarded as evasive latency (EL) and it lasted for four days. And space exploration was carried out on the 5th day, the platform was removed, and time for rats to locate the platform quadrant was recorded as space exploration time (SET).

### 2.6. Surgery

Rats were anesthetized with 4 mL/kg 10% chloral hydrate and mounted on the operating desk after abrosia for 24 h. The heart was exposed and then 150–200 mL 0.01 M PBS was implanted quickly from aorta on the apex cordis, while scissoring the auricula dextra and implanting 150–200 mL 4% paraformaldehyde through aorta slowly until clear fluid came out from auricula dextra. After that, they were beheaded in brain and the hippocampuswas isolated on the cryostage, and then it was fixed in 4% paraformaldehyde for 6–8 h and kept in ice saline (2.5 mL/g) after infusion and limbs stiffness.

### 2.7. Blood Test

The blood in serum tubes was centrifuged and the levels of fasting plasma glucose (FPG), fasting insulin (FINS), glycated hemoglobin (HbAlc), total cholesterol (TC), triglyceride (TG), high-density lipoprotein-cholesterol (HDL-C), and low-density lipoprotein-cholesterol (LDL-C) were tested, respectively, as previous study described [10]. The homeostasis model assessment of insulin resistance was calculated as follows:

(1) $$\begin{array}{c} \left(\text{HOMA-IR}\right)=\frac{\text{FPG}\times \text{FINS}}{22.5}\\ \left(\text{ISI}\right)=\ln\left[\frac{1}{\text{FPG}\times \text{FINS}}\right]\\ \left(\text{HOMA-B}\right)=20\times \frac{\text{FINS}}{\text{FPG}-3.5}. \end{array}$$

### 2.8. ELISA Experiments

One side of hippocampus was sampled for enzyme-linked immunosorbent assay. The tissue was ground in ice saline by refiner before it was centrifuged for 10000 r/min 10 min. Supernatant was collected in −80°C until the content of CORT was measured by ELISA kit (R&D Systems, USA) following the manufacturer's protocol. Samples were added to Microelisa Stripplate and incubated in 37°C for 30 min before 50 *μ*L HRP-conjugate reagent was added, which followed by 30 min incubation at 37°C. And then, chromogen solution and stop solution were added before the optical density value was measured at 450 nm. In the meantime, the standard curve was made. Moreover, the level of CORT in blood plasma was measured by CORT ELISA kit (R&D Systems, USA).

### 2.9. Histopathology Experiment

The brain was removed surgically and fixed in 4% formalin before the tissue was dehydrated, paraffin-embedded, and sliced into section. After that, HE stain was performed and then observed under light microscope.

### 2.10. Western Blotting Experiments

The other side of hippocampus was applied for western blotting (Western Blotting Kit, Beyotime Institute of Biotechnology, China). The tissue was homogenized in cell lysis buffer (NP-40 lysis buffer) with protease inhibitor and it was ground by refiner before supernatant was kept in −20°C after centrifuge. The protein was electrophoretically resolved on 10% SDS-polyacrylamide gels and then transferred to nitrocellulose membranes at 100 mA for 2.5 h. After that, the membranes were blocked in skimmed milk for 1 h at room temperature and overnight at 4°C in anti-11*β*-HSD1 (1 : 1000; Cell Signaling Technology, USA), anti-GR (1 : 1000; Cell Signaling Technology, USA), and anti-*β*-actin (1 : 800; Cell Signaling Technology, USA), receptively. Then the membrane was incubated with HRP antibody (Boster Co., Wuhan, China) at dilution of 1 : 1000. Finally, the membranes were observed by the use of enhanced chemiluminescence kit (ECL, Amersham).

### 2.11. Immunohistochemical Experiments

The brain was performed for immunohistochemical experiment. After the tissues were dehydrated, embedded in paraffin, and sliced into sections, the slides were incubated in 3% hydrogen peroxide for 10 min and heated in 0.01 M citrate buffer. After that, they were incubated with the goat serum sealing fluid for 20 min and covered with 50 *μ*L anti-rabbit, which followed by 4°C overnight. Then, the sections were rewarmed at 37°C for 45 min and incubated with SABC for 30 min, which followed by DAB coloration under the microscope and the sections were redyed with hematoxylin (2 min). Finally, dehydration, hyalinization, and mounting were performed and the sections were observed under high magnification (×400) with picture taken.

### 2.12. Statistics

All the data were based on SPSS16.0 and analyzed by one-way analysis of variance (ANOVA), *t*-test with two-side test. A level of *P* < 0.05 was set as statistically significant.

## 3. Results

### 3.1. ZGJTJY Helps to Increase the State of Behavioral Activity

**Figure 1:**
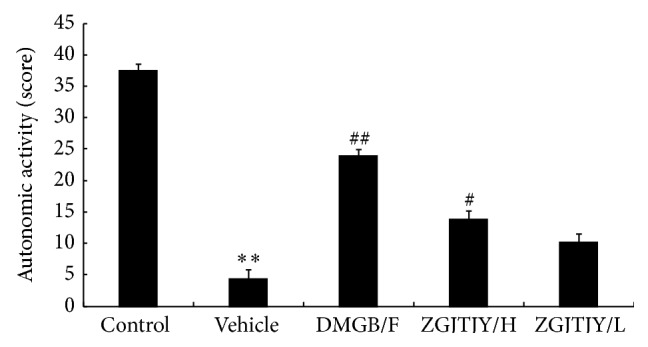
ZGJTJY increases the activity of rats with diabetes-related depression. The autonomic activity was recorded by counting horizontal movement and vertical movement in open field test. ^*^ *P* < 0.05 compared with control, ^**^ *P* < 0.01 compared with control; ^#^ *P* < 0.05 compared with vehicle; ^##^ *P* < 0.01 compared with vehicle.

The mobility of different groups is presented in Figure 1.

The activity in vehicle group was obviously lower than that in control group. However, there was a significant increase of mobility in DMGB/F group from 4.38 ± 1.36 to 23.94 ± 1.02. Moreover, the treatment of ZGJTJY witnessed a positive relationship between its dosages where the activities in ZGJTJY/H group rise from 23.94 ± 1.02 to 13.77 ± 1.29, while from 4.38 ± 1.36 to 10.21 ± 0.98 in ZGJTJY/L group.

### 3.2. Capability of Learning and Memory Was Increased by Treatment of ZGJTJY

**Figure 2:**
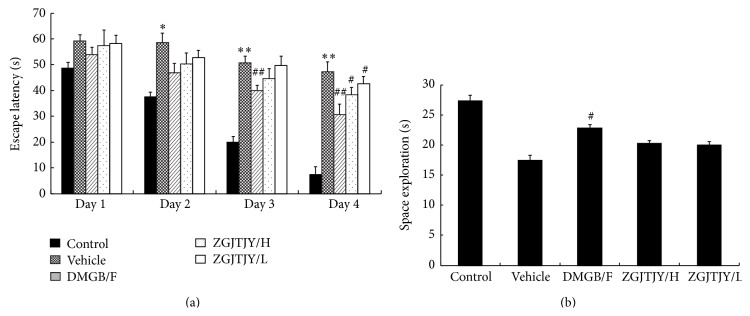
Capability of learning and memory was enhanced after treatment with ZGJTJY. The capabilities of learning were measured by (a) place navigation and the ability of memory was tested by (b) space exploration. ^*^ *P* < 0.05 compared with control, ^**^ *P* < 0.01 compared with control; ^#^ *P* < 0.05 compared with vehicle; ^##^ *P* < 0.01 compared with vehicle.

The capability of learning and memory was presented in Figure 2.

During the 4 days of place navigation, the escape latency (EL) was higher in vehicle when compared with control group, while it has witnessed a fluctuated decrease in DMGB/F and ZGJTJY groups when compared with vehicle group, especially the 4th day on which the treatment effects were significant where EL was declining from 47.22 S ± 3.86 to 38.32 S ± 2.81 in ZGJTJY/H group. Moreover, there was a positive correlation in the dosage of ZGJTJY.

The space exploration demonstrated that the time spent in target quadrant was shorter in vehicle when compared with control group, while it was increased in DMGB/F and ZGJTJY groups. Noticeably, the treatment effect in high dose of ZGJTJY was more efficient than that in the low dose.

### 3.3. The Disorder in Blood Glucose and the Relative Indexes Was Relieved by ZGJTJY

**Figure 3:**
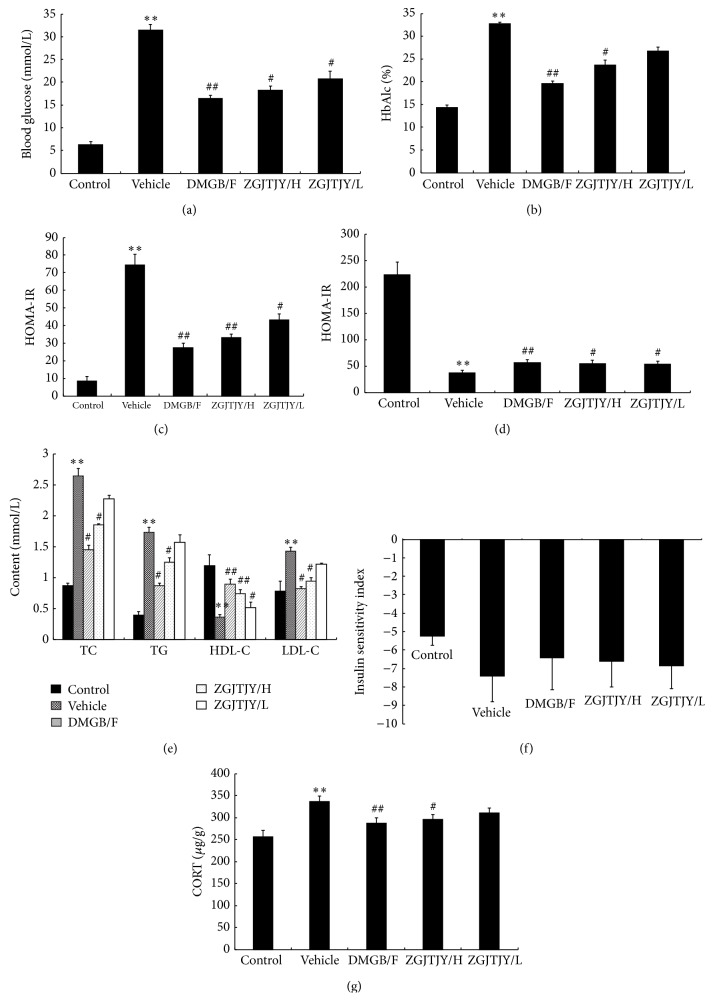
The disorder in blood glucose and the relative indexes in blood was relieved by DMGB/F and ZGJTJY and there was a positive relationship between the dosages of ZGJTJY. The levels of the relative indexes in blood were tested by specific kits, respectively, except the level of CORT which was measured by ELISA. ^*^ *P* < 0.05 compared with control, ^**^ *P* < 0.01 compared with control; ^#^ *P* < 0.05 compared with vehicle; ^##^ *P* < 0.01 compared with vehicle.

The relative indexes in glucose, blood lipid, and insulin are presented in Figure 3.

The levels of blood glucose (Figure 3(a)) and HbAlc (Figure 3(b)) were significantly higher in vehicle group accompanied with severe dysfunction of HOMA-IR (Figure 3(c)) and HOMA-B (Figure 3(d)), and abnormal blood lipid indexes (lipid triad, Figure 3(e)) were performed when compared with control group as well. However, there was a significant treatment influence in DMGB/F and ZGJTJY groups where the levels of blood glucose (Figure 3(a)) and HbAlc (Figure 3(b)) were decreased, the serious HOMA-IR (Figure 3(c)) was relieved, and HOMA-B (Figure 3(d)) was slightly improved. Moreover, the lipid triad has witnessed a back to normal tendency when TC, TG, and LDL-C (Figure 3(e)) were increased and HDL-C has gone down. And the disorder in insulin sensitivity index (Figure 3(f)) was relieved by DMGB/F and ZGJTJY. Moreover, the level of CORT (Figure 3(g)) in blood plasma was obviously high in vehicle, while it was decreased after treatment with DMGB/F and ZGJTJY. Noticeably, there was a positive relationship occurring in ZGJTJY groups where the therapeutic effects were obvious in the high dose.

### 3.4. ZGJTJY Helps to Recover the Damages in Hippocampus Which Are Insulted by Diabetes-Related Depression

**Figure 4:**
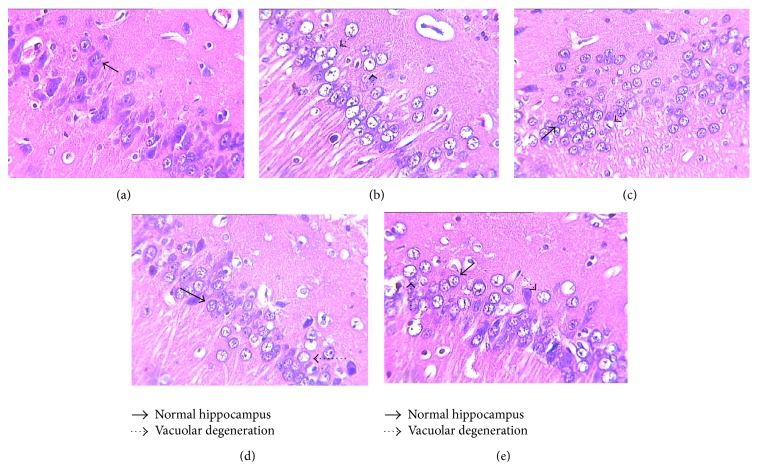
Cytoprotection was obvious in ZGJTJY group where the normal hippocampus appeared to increase. The morphology changes in hippocampus were observed by HE stain. (a) Control; (b) vehicle; (c) DMGB/F; (d) ZGJTJY/H; (e) ZGJTJY/L.

The alternations in hippocampus are present in Figure 4.

In control group, normal hippocampus cones were observed, while dark cell plasma and vacuolar degeneration occurred in vehicle. The recovery of hippocampus in DMGB/F and ZGJTJY groups was obvious. Compared with low level of ZGJTJY, the vacuolar degeneration and cell swelling were significantly decreased in the high level. Thus, there is positive relationship between the dosage of ZGJTJY and cytoprotection.

### 3.5. ZGJTJY Leads to a Decrease of CORT in Hippocampus

**Figure 5:**
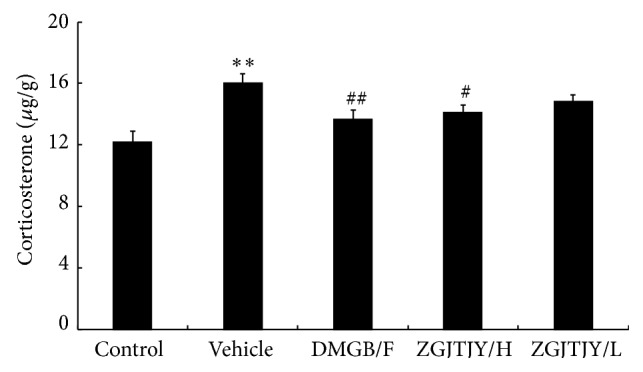
The expression of CORT was inhibited by treatment of ZGJTJY. The CORT expression in hippocampus was measured by ELISA kit following the manufacturer's protocol. ^*^ *P* < 0.05 compared with control; ^**^ *P* < 0.01 compared with control; ^#^ *P* < 0.05 compared with vehicle, ^##^ *P* < 0.01 compared with vehicle; *n* = 8, $\overset{-}{x}\pm s$.

The content of CORT in hippocampus is presented in Figure 5.

The expression of CORT in vehicle (Figure 5) was obviously high when compared with the control. However, the expression was decreased significantly in DMGB/F and ZGJTJY/H groups, while the treatment effect in low level of ZGJTJY was slight. The result indicated that there is a positive correlated relationship between the decline of CORT and the dosage of ZGJTJY.

### 3.6. Downregulation of 11*β*-HSD1 Was Caused by ZGJTJY in Hippocampus

**Figure 6:**
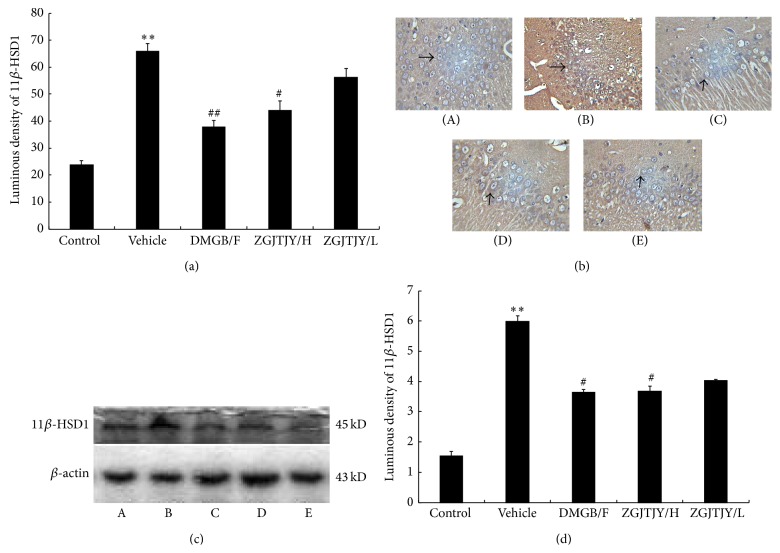
Inhibition of 11*β*-HSD1 was caused by ZGJTJY. The protein level was measured by immunohistochemistry (a, b) and western blotting (c, d). ^*^ *P* < 0.05 compared with control, ^**^ *P* < 0.01 compared with control; ^#^ *P* < 0.05 compared with vehicle; ^##^ *P* < 0.01 compared with vehicle; *n* = 8, $\overset{-}{x}\pm s$. (A) Control group; (B) vehicle; (C) DMGB/F; (D) ZGJTJY/H; (E) ZGJTJY/L.

The protein level of 11*β*-HSD1 was measured by immunohistochemistry (Figures 6(a) and 6(b)) and western blotting (Figures 6(c) and 6(d)). The result (Figure 6(b)) shows that 11*β*-HSD1-like positive immunoreactivity neuron has become stronger in vehicle group when compared with control. Thus the luminous density of 11*β*-HSD1 positive cell was significantly higher in group vehicle (Figure 6(a)). However, there is an obvious decline of 11*β*-HSD1 in DMGB/F (*P* < 0.05, Figure 6(a)) and ZGJTJY groups. Furthermore, a positive correlation was found between the decrease of 11*β*-HSD1 and the dose of ZGJTJY where the high dose of ZGJTJY witnessed a dramatic decrease of 11*β*-HSD1 (Figure 6(a)). As shown in Figures 6(c) and 6(d), the western blotting test indicates that the changes between different groups nearly shared the same tendency with immunohistochemistry.

### 3.7. ZGJTJY Leads to an Increase of GR in Hippocampus

**Figure 7:**
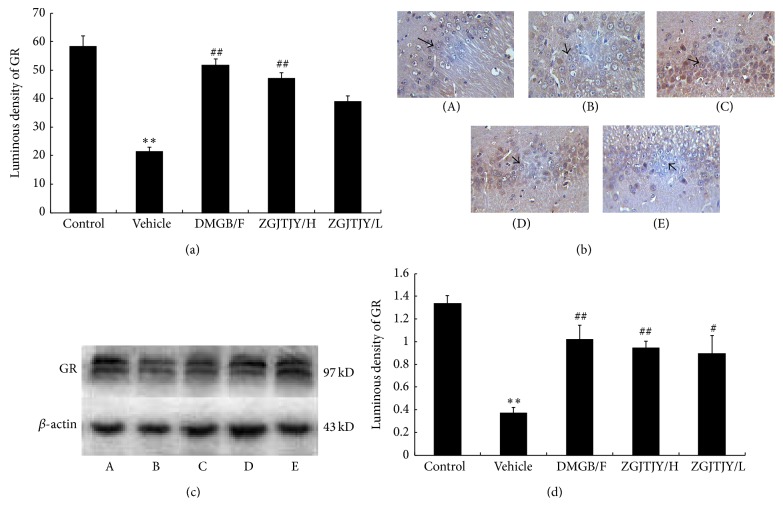
The expression of GR was restrained by ZGJTJY. The protein level of GR was measured by immunohistochemistry (a, b) and western blotting (c, d). ^*^ *P* < 0.05 versus control, ^**^ *P* < 0.01 versus control; ^#^ *P* < 0.05 versus vehicle, ^##^ *P* < 0.01 versus vehicle; *n* = 8, $\overset{-}{x}\pm s$. (A) Control group; (B) vehicle; (C) DMGB/F; (D) ZGJTJY/H; (E) ZGJTJY/L.

The protein level of GR in hippocampus was measured by immunohistochemistry and western blotting. The results indicate that the vehicle (Figure 7(b)) group witnessed a significant decrease in GR-like positive immunoreactivity neuron when compared with control group (*P* < 0.05) that means the expression of GR was restrained by diabetes-related depression. The content of GR was increased in DMGB/F and ZGJTJY groups. Noticeably, there is a positive correlation between the upregulation of GR and the dose of ZGJTJY. A remarkable increase was seen in the high dose of ZGJTJY (Figure 7(a)). When it comes to western blotting (Figures 7(c) and 7(d)), the trend of GR was almost the same as the result of immunohistochemistry where there is a positive relationship between the increase of GR and the dosage of ZGJTJY as well.

## 4. Discussion

In this study, we found that ZGJTJY can effectively lower blood glucose level in diabetes-related depression rats (Figure 3) and improve their depressive behavior (Figures 1 and 2). CORT can not only regulate blood glucose variation, but also damage the hippocampus and lead to depressive behavior because of its high concentration. Our study found that the disordered blood glucose and behavior attributed to the abnormal increase of CORT in diabetes-related depression rats (Figure 3). Although a lot of studies have examined the relationship between CORT and blood glucose, few have observed the association between CORT and hippocampus. In this study, we speculated that the deregulated expression of 11*β*-HSD1 in hippocampus (Figure 6) can lead to increased CORT activity, while a decreased expression of glucocorticoid receptor (Figure 7) leads to a situation that CORT is unable to correct the HPA axis disorders through negative feedback and then causes CORT accumulation and damages the neurons. Those might be the important reasons to explain the hippocampal damage in diabetes-related depression. ZGJTJY could relieve hippocampal damage by improving CORT regulation through inhibiting the expression of 11*β*-HSD1 and increasing GR.

The methods of establishing the diabetes-related depression in the study consist of high fat diet, STZ injection, and 28 days of unpredictable chronic mild stress, which are reliable and stable. The chronic mild stress utilized in this research not only decreases the stimulus intensity, but better simulates the living pressure during the daily life as well. Consequently, physiological changes may occur in the rats after a series of stimulations; moreover, the randomly applied chronic mild stress made rats difficult to adopt, which is conducive to establishing the model. Although there are some other methods to establish a depression model, most of those approaches are inappropriate to build a diabetes-related depression. For instance, brain injury models including olfactory bulb resection model contribute to critical damages which are difficult for diabetes rats to bear. Force swim and tail suspension test with a short time model maintenance are instable in this study, which made the results not credible.

An increased HPA axis activity was found in diabetes, which then leads to secretion of GC in adrenal glands with an increase of CORT in blood circulation and local brain tissue. The elevated cortisol levels in the hippocampal neurons cause neurotoxicity and affect the cognitive function, which then results in depression. GC relied on the activation of 11*β*-HSD1 to pose effects on target tissues. 11*β*-HSD1, a NADP(H)-dependent with a low affinity dehydrogenation/oxidoreductase enzyme, was a stimulator that transforms the nonactive GC, namely, cortisone (human) and 11*β*-dehydrogenase corticosterone (rodents), into active cortisol (human) and corticosterone (rodents), respectively, and then it amplifies the local effect of GC [21, 22]. The enzyme is highly expressed in hippocampus which located in paraventricular nucleus of hypothalamus and cerebral cortex in the central nervous system. 11*β*-HSD1 expression was increased in diabetic rat [19]. Inactivated 11*β*-dehydrogenase corticosterone was circulated to hippocampus and stimulated by high expression of 11*β*-HSD1 into corticosterone, further elevating corticosterone levels in local brain [19, 20], which then leads to the neuron damage and necrosis of hippocampus and reduces the number of neurons. Therefore spatial learning and memory declined. And eventually diabetes-related depression occurred. However, after being regulated by ZGJTJY, the expression of CORT was increased and 11*β*-HSD1 was inhibited in which the effects were related to the dosage of the prescription.

Hippocampus has a wealth of glucocorticoid receptor (GR) and mineralocorticoid receptor (MR). The affinity between GC and MR is almost saturated (>80%) which was ten times as much as it is between GC and GR; the affinity fluctuated from 10% to 90% randomly. Thus, the concentration of GC was the major element that affects the affinity with GR [18, 23]. Previous study found that the concentration of GC in diabetic rats was increased, but the expression of GR was reduced. And those may be due to the decreased number of neurons which are then attributed to the partially increased active glucocorticoids and hippocampal damage [24]. Elevated hippocampal GC combined with GR leads to negative feedback inhibition of the HPA axis under physiological condition [25, 26]. Reduced GR in the hippocampus decreases the inhibition of the HPA axis [27], which then stops the HPA axis circadian rhythm as a result, while stress responses continued, as well as increased secretion of GC level [28]. On one hand, increased GC circulated to the hippocampus, and then it was amplified by 11*β*-HSD1 to further increase the level of corticosterone, which eventually causes vicious cycle and aggravates hippocampal damage [29]. On the other hand, the upregulation of blood corticosterone contributes to a high level of blood glucose in that it was the counterregulatory factor of insulin to prevent insulin secretion. High level of blood glucose, followed by neuronal apoptosis, deteriorates hippocampal damage and leads to depressive disorder. In this study, the expression of GR was significantly reduced in diabetes mellitus with depression, while it was back to normal after treatment with ZGJTJY.

Currently, diabetes-related depression is still at clinical research stage, and there is no specific drug which is usually with negative side effects. However, traditional Chinese medicine is usually based on the overall aspect and it is extraordinarily efficient in complication therapy. ZGJTJY was based on the strength of Zuogui Wan from Jingyue Zhang, and it has shown better hypoglycemic and antidepressant effects. By this experiment studying, ZGJTJY can significantly improve blood glucose and depressive behavior in rats with diabetes-related depression, as well as reducing corticosterone levels, and inhibit the expression of 11*β*-HSD1 in the hippocampus and increase the expression of GR. In order to have a further insight of ZGJTJY, our group intends to perform further study on the effect of ZGJTJY on cortisol regulation of diabetes-related depression rats in hippocampus.
